# B7-H3 Augments Inflammatory Responses and Exacerbates Brain Damage via Amplifying NF-κB p65 and MAPK p38 Activation during Experimental Pneumococcal Meningitis

**DOI:** 10.1371/journal.pone.0171146

**Published:** 2017-01-31

**Authors:** Xuqin Chen, Yan Li, Siobhan Blankson, Min Liu, Danping Huang, H. Paul Redmond, Jing Huang, Jiang Huai Wang, Jian Wang

**Affiliations:** 1 Department of Neurology, Children’s Hospital of Soochow University, Suzhou, China; 2 Pediatric Research Institute of Soochow University, Suzhou, China; 3 Department of Academic Surgery, University College Cork, Cork University Hospital, Cork, Ireland; Southern Medical University, CHINA

## Abstract

The costimulatory protein B7-H3 has been shown to play a contributory role in the development and progression of experimental pneumococcal meningitis by augmentation of the innate immunity-associated inflammatory response via a TLR2-dependent manner. This study aimed to clarify the component(s) of TLR2-mediated signal transduction pathways responsible for B7-H3-augmented inflammatory response and subsequent brain damage during experimental pneumococcal meningitis. Administration of B7-H3 did not augment expression of TLR2 and other TLR2 upstream components, but led to an enhanced formation of MyD88-IRAK immunocomplex in the brain of *S*. *pneumoniae*-infected mice. Furthermore, B7-H3 substantially augmented *S*. *pneumoniae*-induced activation of TLR2 downstream NF-κB p65 and MAPK p38 pathways in the brain of *S*. *pneumoniae*-infected mice. Notably, blockage of NF-κB p65 and/or MAPK p38 with their specific inhibitors strongly attenuated B7-H3-amplified inflammatory response with significantly reduced proinflammatory cytokine and chemokine production, and markedly ameliorated B7-H3-exacerbated disruption of blood-brain barrier and severity of disease status in *S*. *pneumoniae*-infected mice. These results indicate that targeting NF-κB p65 and/or MAPK p38 may represent a promising therapeutic option for amelioration of overwhelming inflammatory response-associated brain injury frequently observed during pneumococcal meningitis.

## Introduction

Bacterial meningitis is among the top 10 causes of infection-related deaths worldwide and many survivors suffer from permanent neurological sequelae [1]. Bacterial meningitis caused by *Streptococcus pneumoniae* (*S*. *pneumoniae*) is the principal and most frequent etiological agent for bacterial meningitis in humans and accounts for more than half of all cases [2,3]. Pneumococcal meningitis remains a life-threatening infectious disease in both adults and children, and causes death in approximately 25% of cases and neurological sequelae in nearly half of survivors despite targeted antibiotic therapy, adjunctive treatment with steroids, and supportive intensive care [4–7]. It has been well established that the poor outcome of pneumococcal meningitis is often associated with the development of intracranial complications such as brain edema and cerebrovascular damage [8,9]. Mounting evidence has shown that the development and progression of pneumococcal meningitis and its intracranial complications are not only due to an uncontrolled bacterial growth in the central nervous system (CNS), but also dependent largely on the host innate immunity-initiated inflammatory response through the recognition of pathogen-associated molecular patterns (PAMPs) on the invaded microbial pathogens by the pattern recognition receptors (PRRs) such as toll-like receptor 2 (TLR2) and its adaptor protein myeloid differentiation factor 88 (MyD88) [10–12]. *S*. *pneumoniae* infection of the meninge often generates an overwhelming inflammatory reaction via both TLR2- and MyD88-dependent production of proinflammatory cytokines and chemokines [11–13]. Although the inflammatory response triggered by *S*. *pneumoniae* infection normally helps to eradicate the invaded microbial pathogens from the CNS, a persistent and/or amplified activation of this reaction with the excessive production of proinflammatory cytokines in the CNS may cause severe damage to the brain, thus contributing to a frequently unfavorable outcome during the development of pneumococcal meningitis [8,10,14].

B7-H3 is a newly discovered member of the B7 costimulatory protein superfamily and has been identified in both humans and mice by sharing ∼88% amino acid sequence identity [15,16]. Accumulated evidence supports the notion that B7-H3 functions as both a T cell costimulator and coinhibitor, thus possessing a contrasting role in regulation of Ag-specific T cell-mediated immune responses [16–19]. More recently, B7-H3 has been shown to participate in the innate immunity-associated inflammatory response. B7-H3 is inducible in human monocytes/macrophages and dendritic cells upon inflammatory cytokine stimulation [16,20]. Our recent work demonstrated an inflammation-based action of B7-H3 by augmenting both the TLR2 agonist bacterial lipoprotein (BLP)- and the TLR4 agonist lipopolysaccharide (LPS)-stimulated nuclear factor-kappaB (NF-κB) activation and proinflammatory cytokine production in monocytes/macrophages [21]. Patients diagnosed with bacterial meningitis displayed significantly elevated soluble B7-H3 (sB7-H3) in the circulation and cerebrospinal fluid (CSF), and levels of sB7-H3 in these patients correlated closely with the intensity of their infectious inflammatory process in the CNS [22]. In a murine model of pneumococcal meningitis, we found that B7-H3 strongly enhanced *S*. *pneumoniae*-stimulated proinflammatory cytokine and chemokine expression in the brain via a TLR2-dependent mechanism, and this B7-H3-amplified inflammatory response further exacerbated *S*. *pneumoniae*-induced disruption of blood-brain barrier (BBB) integrity and brain damage [13,23], indicating that B7-H3 participates in the development of pneumococcal meningitis via augmentation of the innate immunity-associated inflammatory response.

In the present study, we investigated the impact of B7-H3 on activation of TLR2 signaling in a murine model of pneumococcal meningitis, in an attempt to clarify the component(s) of TLR2-mediated signal transduction pathways responsible for B7-H3-induced amplification of inflammatory responses and subsequent brain damage. We reported here that administration of B7-H3 resulted in an enhanced formation of MyD88-IL-1 receptor-associated kinase (IRAK) immunocomplex and augmented activation of TLR2 downstream NF-κB p65 and mitogen-activated protein kinase (MAPK) p38 in *S*. *pneumoniae*-infected mice. Remarkably, blockage of either NF-κB p65 or MAPK p38 attenuated B7-H3-amplified inflammatory response with significantly reduced proinflammatory cytokine and chemokine production, and ameliorated B7-H3-exacerbated BBB disruption and brain damage in *S*. *pneumoniae*-infected mice.

## Materials and Methods

### Reagents, antibodies, and bacteria

Recombinant mouse B7-H3 was purchased from R&D Systems (Minneapolis, MN, USA). Antibodies (Abs) that recognize TLR2, MyD88, and IRAK-1 were purchased from Santa Cruz Biotechnology (Santa Cruz, CA, USA) and Abcam (Cambridge, MA, USA), respectively. Abs that recognize NF-κB p65, phospho-p65 at Ser536, MAPK p38, and phospho-p38 at Thr180/Tyr182 were purchased from Santa Cruz Biotechnology and Cell Signaling Technology (Beverly, MA, USA), respectively. The MAPK p38 inhibitor SB203580 and NF-κB p65 inhibitor PDTC were obtained from Cell Signaling Technology and Merk Millipore (Billerica, MA, USA), respectively. All other chemicals, unless indicated, were from Sigma-Aldrich (St. Louis, MO, USA).

Gram-positive *S*. *pneumoniae* type 3 was obtained from American Type Culture Collection (ATCC, Manassas, VA, USA). Bacteria were cultured at 37°C in trypticase soy broth (Merck, Darmstadt, Germany), harvested at the mid-logarithmic growth phase, washed twice, and resuspended in PBS. The concentration of resuspended bacteria was determined and adjusted spectrophotometrically at 550 nm.

### Mice and pneumococcal meningitis

Pyrogen-free, 8- to 10-week old male Balb/c mice were purchased from Slac (Shanghai, China). Mice were housed in barrier cages under controlled environmental conditions (12/12 hrs of light/dark cycle, 55% ± 5% humidity, 23°C) in the Pediatric Research Institute of Soochow University and had free access to standard laboratory chow and water. Animals were fasted 12 hrs before experiments and allowed water *ad libitum*. All animal studies were ethically approved by the institutional animal care and use committee at Soochow University and complied with the animal welfare act. The methods applied in this study were carried out in accordance with the approved guidelines.

Age- and weight-matched male Balb/c mice were anesthetized by intramuscular injection of 150 μl ketamine/xylazine admixture (150 μl ketamine plus 150 μl xylazine made up to 1 ml with 0.9% saline). Pneumococcal meningitis was induced by intracerebral ventricular injection of 15 μl sterile PBS containing 0.75×10^7^ CFU/ml *S*. *pneumoniae* (SP) into the lateral ventricle as described previously [13,23].

### Experimental groups and assessment of the clinical disease status

Eight- to ten-week old male Balb/c mice (n = 192 in total) were randomized into one of the following four experimental groups (n = 30 per group) and each mouse received an intracerebral ventricular injection of 15 μl in total: 1) mice in the control group injected with 15 μl PBS; 2) mice in the B7-H3 group injected with 15 μl PBS containing 2.5 μg B7-H3; 3) mice in the SP group injected with 15 μl PBS containing 0.75×10^7^ CFU/ml *S*. *pneumoniae*; 4) mice in the SP plus B7-H3 group injected with 7.5 μl PBS containing 1.5×10^7^ CFU/ml *S*. *pneumoniae* and 7.5 μl PBS containing 2.5 μg B7-H3. For blocking NF-κB p65 and/or MAPK p38, mice were received an intracerebral ventricular injection of 7.5 μl PBS containing equivalent dimethyl sulfoxide (DMSO), the MAPK p38 inhibitor SB203580 (40 μg/mouse), the NF-κB p65 inhibitor PDTC (100 μg/mouse), or SB203580 plus PDTC (40+100 μg/mouse) 1 hr before mice treated with PBS, *S*. *pneumoniae*, or *S*. *pneumoniae* plus B7H3 (n = 24 per group) as described above. The in vivo study was carried out in two separate experiments.

Mice were weighed, allowed to wake up, and evaluated clinically at 6, 18, and 30 hrs after SP infection. The clinical disease status was examined by spontaneous motor activity and body weight loss. The following scores were used to assess spontaneous motor activity of mice as described previously [24,25]: 1, normal motor activity and turned upright in <5 s when put on their back; 2, reduced spontaneous motor activity, but still turned up in <5 s; 3, turned up in >5 s; 4, did not turn up; 5, did not move at all. At the indicated time points after SP infection, mice were sacrificed by CO_2_ inhalation. The brain of each animal was removed, half of the brain was frozen immediately in liquid nitrogen and stored at -80°C for quantitative real-time PCR and ELISA, and the other part of the brain was used for immunoblotting and immunoprecipitation.

### Quantitative real-time PCR

TLR2, MyD88, IRAK-1, toll-interleukin 1 receptor domain containing adaptor protein (TIRAP), TNF receptor-associated factor 6 (TRAF6), TNF-α, IL-1β, IL-6, and MCP-1 mRNA expression was assessed by quantitative real-time PCR. Total RNA was extracted from frozen brain sections using a GeneElute^TM^ mammalian total RNA purification kit (Sigma-Aldrich) and reverse-transcribed into cDNA using the SuperScript first-strand synthesis system (Invitrogen Life Technologies, Paisley, Scotland, UK). Amplification of cDNA was conducted using a LightCycler system (Roche Molecular Biochemicals, Indianapolis, IN, USA) according to the manufacturer’s instructions. The target gene mRNA expression was normalized with the housekeeping gene β-actin. Gene-specific primers used in this study were listed in **Table 1**.

**Table 1:** Gene-specific PCR primers.

	Product length (bp)	Sense	Antisense
Mouse TLR2	231	GCAAACGCTGTTCTGCTCAG	AGGCGTCTCCCTCTATTGTATT
Mouse MyD88	175	TCATGTTCTCCATACCCTTGGT	AAACTGCGAGTGGGGTCAG
Mouse IRAK-1	195	CCACCCTGGGTTATGTGCC	GAGGATGTGAACGAGGTCAGC
Mouse TIRAP	100	CCTCCTCCACTCCGTCCAA	CTTTCCTGGGAGATCGGCAT
Mouse TRAF6	125	AAAGCGAGAGATTCTTTCCCTG	ACTGGGGACAATTCACTAGAGC
Mouse TNF-α	122	CTGAACTTCGGGGTGATCGG	GGCTTGTCACTCGAATTTTGAGA
Mouse IL-1β	116	GAAATGCCACCTTTTGACAGTG	TGGATGCTCTCATCAGGACAG
Mouse IL-6	131	CTGCAAGAGACTTCCATCCAG	AGTGGTATAGACAGGTCTGTTGG
Mouse MCP-1	121	TTAAAAACCTGGATCGGAACCAA	GCATTAGCTTCAGATTTACGGGT
β-actin	211	GTGACGTTGACATCCGTAAAGACC	ATCTGCTGGAAGGTGGACAGTGAG

### ELISA

Brain samples collected at the indicated time points after SP infection were homogenized and centrifuged. The concentrations of proinflammatory cytokines TNF-α, IL-1β, IL-6 and chemokine MCP-1 in brain homogenates were assessed by ELISA (eBioscience, Hatfield, UK) according to the manufacturer’s instructions.

Two endogenous serum abundant proteins albumin and IgG are normally excluded from the brain by the intact BBB. To assess the BBB integrity, albumin and IgG levels in mouse brain homogenates collected at 30 hrs after SP infection were assessed by ELISA (RD Biotech, Besancon, France) (Abnova, Walnut, CA, USA), respectively, according to the manufacturer’s instructions.

### Immunoblotting and immunoprecipitation

At the indicated time points after SP infection, brain samples were collected, homogenized, and lysed on ice in lysis buffer (Cell Signaling Technology), supplemented with 1 mM phenylmethylsulfonyl fluoride (PMSF) and protease inhibitor cocktail (Roche Molecular Biochemicals). The resultant lysates were centrifuged and supernatants containing the cytoplasmic proteins were collected. Protein concentrations were determined using a micro BCA protein assay (Pierce, Rockford, IL, USA). Equal amounts of protein extracts were separated on SDS-polyacrylamide gels and trans-blotted onto polyvinylidene difluoride (PVDF) membranes (Schleicher & Schuell, Dassel, Germany). The membrane was blocked for 1 hr at room temperature with PBS containing 0.05% Tween-20 and 5% nonfat milk, and probed overnight at 4°C with primary Abs. Blots were then incubated with appropriate horseradish peroxidase-conjugated secondary Abs (Cell Signaling Technology) at room temperature for 1 hr, developed with SuperSignal chemiluminescent substrate (Pierce), and captured with LAS-3000 imaging system (Fujifilm, Tokyo, Japan). For immunoprecipitation, equal amounts of extracted protein were incubated with anti-MyD88 Ab overnight at 4°C on a rotator. Thereafter, 50 μl of 50% slurry of pre-washed protein A/G-agarose beads (Pierce) was added to each sample and incubated at 4°C for 2 hrs. The samples were spun briefly and washed three times in the lysis buffer. Loading buffer was added to each sample and boiled for 10 min. The samples were then separated by SDS-polyacrylamide gels, trans-blotted onto PVDF membranes, and probed with anti-IRAK-1 Ab.

### Statistical analysis

All data are presented as the mean ± SD. Statistical analysis was performed with GraphPad software version 5.01 (Prism, La Jolla, CA, USA). Comparisons between groups were carried out using one-way ANOVA, followed by Bonferroni correction when necessary, and Mann-Whitney *U* test, where appropriate. Differences in spontaneous motor activity and body weight loss between groups were analyzed using Kruskal-Wallis test. A *p* value <0.05 was judged statistically significant.

## Results

### B7-H3 fails to augment TLR2 and other upstream component expression, but enhances MyD88-IRAK immunocomplex formation in the brain of S. pneumoniae-infected mice

**Fig 1:**
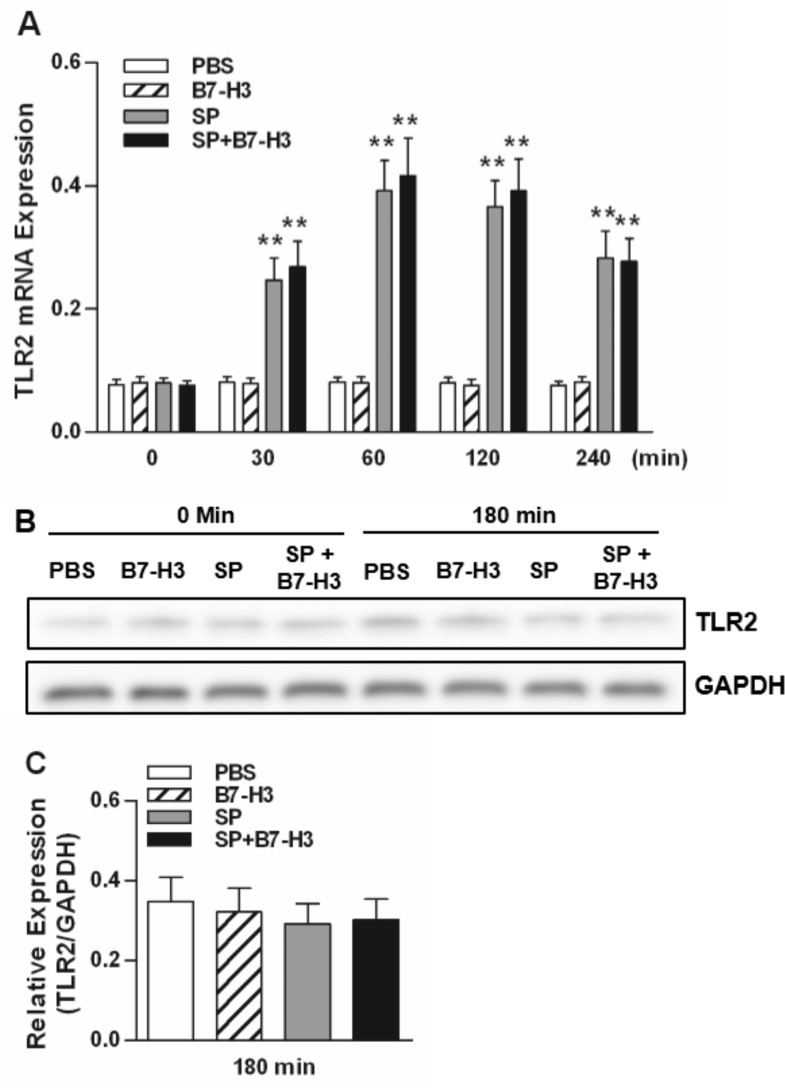
B7-H3 does not augment TLR2 expression in brain tissues of *S*. *pneumoniae*-infected mice. Mice were challenged with PBS as the control, recombinant mouse B7-H3, live *S*. *pneumoniae* (SP), or live *S*. *pneumoniae* plus B7-H3 (SP+B7-H3) via intracerebral ventricular injection as described in the Methods. Brain samples were collected at the indicated time points after challenges. TLR2 mRNA expression was assessed by quantitative real-time PCR (**A**) and data are expressed as mean ± SD of five to six mice per time point and represent two separate experiments. **p<0.01 compared with mice treated with PBS. TLR2 protein expression (**B**) was detected by Western blot analysis and data shown represent one experiment from a total of four separate experiments. Density ratios of TLR2/GAPDH (n = 4) (**C**) were quantified by densitometry analysis.

To ascertain the component(s) of TLR2-mediated signal transduction pathways responsible for B7-H3-amplified inflammatory response during pneumococcal meningitis, we first examined the impact of B7-H3 on expression of TLR2, MyD88, IRAK1, TIRAP, and TRAF6 in brain tissues of *S*. *pneumoniae*-infected mice. Significantly increased mRNA expression of TLR2 in brain homogenates was observed after mice infected with *S*. *pneumoniae* compared with mice treated with PBS (Fig 1A); however, infection of mice with *S*. *pneumoniae* did not enhance protein expression of TLR2 (Fig 1B and 1C). Moreover, administration of B7-H3 was unable to further augment *S*. *pneumoniae*-stimulated TLR2 expression, as shown in mice challenged with a combination of *S*. *pneumoniae* and B7-H3 in comparison with mice challenged with *S*. *pneumoniae* alone (Fig 1).

**Fig 2:**
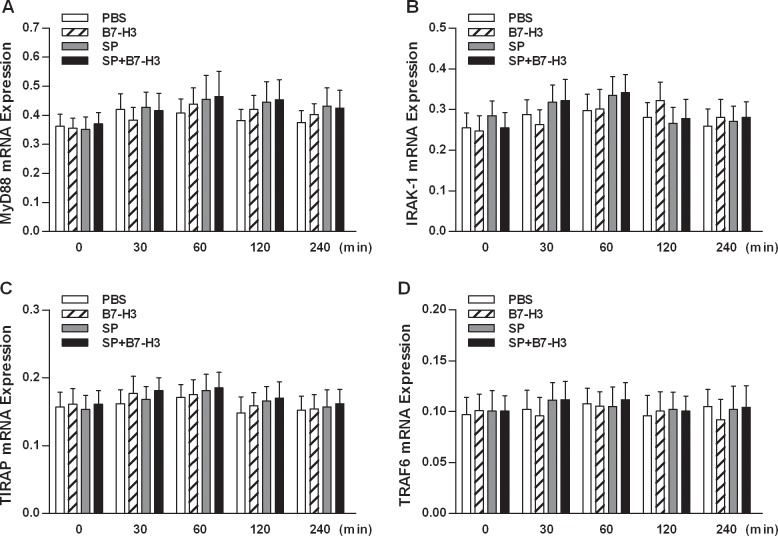
Neither *S*. *pneumoniae* nor B7-H3 enhances mRNA expression of MyD88, IRAK-1, TIRAP, and TRAF6 in brain tissues of *S*. *pneumoniae*-infected mice. Mice were challenged with PBS as the control, recombinant mouse B7-H3, live *S*. *pneumoniae* (SP), or live *S*. *pneumoniae* plus B7-H3 (SP+B7-H3) via intracerebral ventricular injection as described in the Methods. Brain samples were collected at the indicated time points after challenges and MyD88 (**A**), IRAK-1 (**B**), TIRAP (**C**), and TRAF6 (**D**) mRNA expression was assessed by quantitative real-time PCR. Data are expressed as mean ± SD of five to six mice per time point and represent two separate experiments.

**Fig 3:**
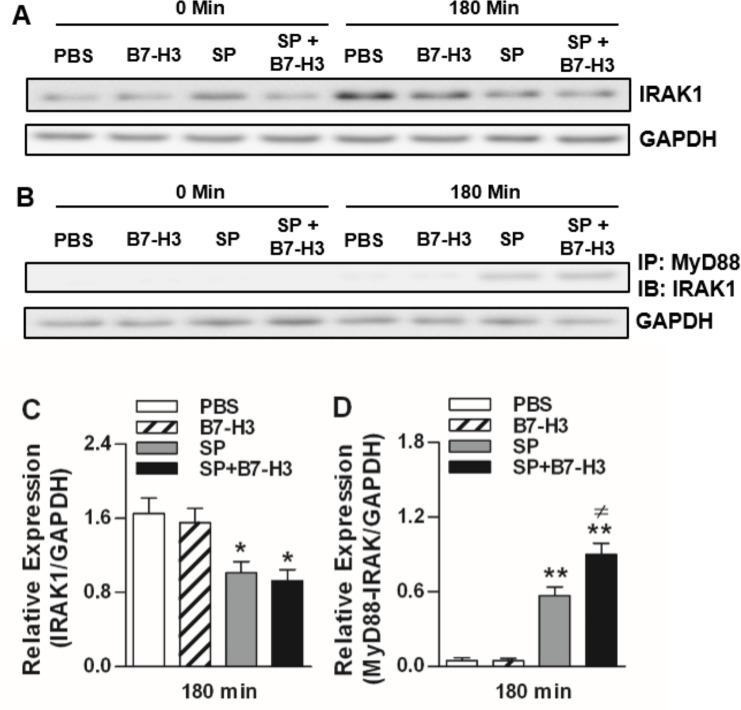
Administration of B7-H3 causes increased MyD88-IRAK immunocomplex formation in brain tissues of *S*. *pneumoniae*-infected mice. Mice were challenged with PBS as the control, recombinant mouse B7-H3, live *S*. *pneumoniae* (SP), or live *S*. *pneumoniae* plus B7-H3 (SP+B7-H3) via intracerebral ventricular injection as described in the Methods. Brain samples were collected at the indicated time points after challenges. IRAK-1 protein expression was detected by Western blot analysis (**A**) and MyD88-IRAK immunocomplex formation was detected by immunoprecipitation (**B**). Data shown represent one experiment from a total of three or four separate experiments. Density ratios of IRAK1/GAPDH (n = 4) (**C**) and MyD88-IRAK/GAPDH (n = 3) (**D**) were quantified by densitometry analysis. *p<0.05, **p<0.01 compared with mice treated with PBS, ^≠^*p*<0.05 compared with mice treated with SP alone.

The recruitment of upstream components of TLR2 signaling including MyD88, and IRAK1, in particular their immunocomplex formation, after engagement of TLR2 with its bacterial ligands plays a crucial role in TLR2-mediated intracellular signal transduction pathways and subsequent initiation of inflammatory responses [26–30]. We next assessed the expression of these upstream components in brain tissues of mice challenged with *S*. *pneumoniae* alone or *S*. *pneumoniae* and B7-H3 in combination. As shown in Fig 2, neither *S*. *pneumoniae* nor *S*. *pneumoniae* in combination with B7-H3 affected the mRNA expression of MyD88, IRAK1, TIRAP, and TRAF6 at all time points measured. However, infection with *S*. *pneumoniae* or *S*. *pneumoniae* in combination with B7-H3 resulted in substantially reduced protein expression of IRAK1 (Fig 3A and 3C). Furthermore, *S*. *pneumoniae* infection led to an enhanced MyD88-IRAK immunocomplex formation, which was further augmented by *S*. *pneumoniae* in combination with B7-H3 (*p*<0.05 versus mice challenged with *S*. *pneumoniae* alone) (Fig 3B and 3D). B7-H3 alone had no effect on IRAK1 expression and its complex formation with MyD88 (Fig 3). These results indicate that B7-H3 amplifies *S*. *pneumoniae*-activated TLR2 upstream signaling pathways, in particular the formation of MyD88-IRAK immunocomplex.

### B7-H3 augments the activation of TLR2 downstream signaling pathways, NF-κB p65 and MAPK p38, in the brain of S. pneumoniae-infected mice

**Fig 4:**
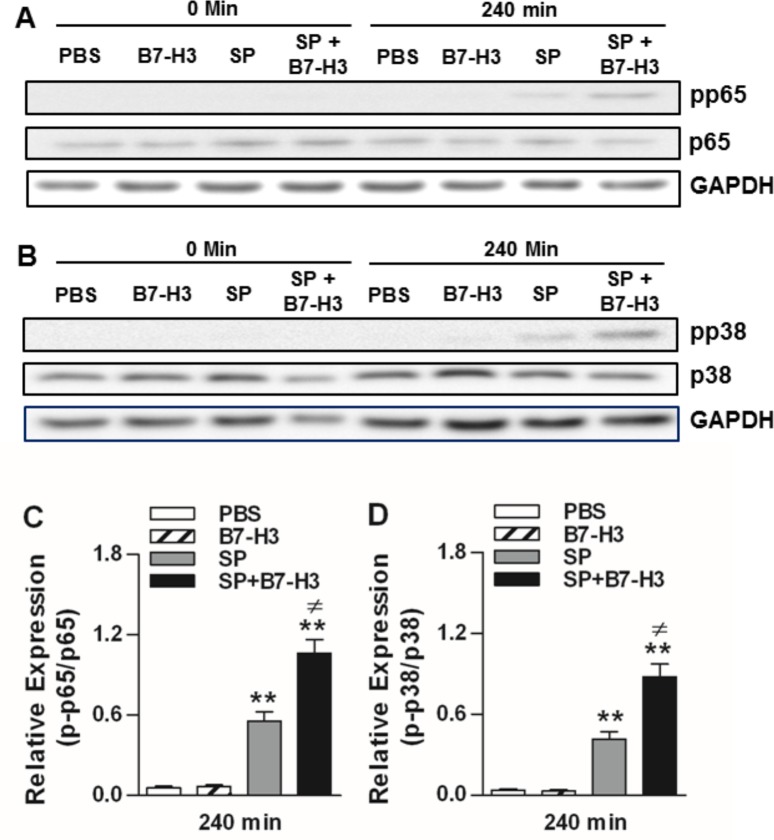
Administration of B7-H3 up-regulates phosphorylation of NF-κB p65 and MAPK p38 in brain tissues of *S*. *pneumoniae*-infected mice. Mice were challenged with PBS as the control, recombinant mouse B7-H3, live *S*. *pneumoniae* (SP), or live *S*. *pneumoniae* plus B7-H3 (SP+B7-H3) via intracerebral ventricular injection as described in the Methods. Brain samples were collected at the indicated time points after challenges. Phosphorylated p65 at Ser536 (**A**), total p65 (**A**), phosphorylated p38 at Thr180/Tyr182 (**B**), and total p38 (**B**) was detected by Western blot analysis. Data shown represent one experiment from a total of four separate experiments. Density ratios of p-p65/p65 (n = 4) (**C**) and p-p38/p38 (n = 4) (**D**) were quantified by densitometry analysis. **p<0.01 compared with mice treated with PBS, ^≠^*p*<0.05 compared with mice treated with SP alone.

We next examined the impact of B7-H3 on activation of NF-κB p65 and MAPK p38, two common downstream pathways for TLR2 signaling [26,27,31], in *S*. *pneumoniae*-infected mice. Infection with *S*. *pneumoniae* induced activation of the NF-κB 65 pathway, as demonstrated by substantially enhanced phosphorylation of p65 at Ser536 in brain homogenates after mice challenged with *S*. *pneumoniae* (Fig 4A and 4C). Similar activation of the MAPK p38 pathway was also observed in *S*. *pneumoniae*-challenged mice, with markedly enhanced phosphorylation of p38 at Thr180/Tyr182 in brain tissues upon *S*. *pneumoniae* infection (Fig 4B and 4D). Notably, B7-H3 itself did not affect phosphorylation of NF-κB p65 and MAPK p38 (Fig 4). However, further substantially increased levels of phosphorylated p65 (Fig 4A and 4C) and p38 (Fig 4B and 4D) were evident in mice challenged with a combination of *S*. *pneumoniae* and B7-H3 (*p*<0.05 versus mice challenged with *S*. *pneumoniae* alone), indicating that administration of B7-H3 results in augmented activation of both NF-κB p65 and MAPK p38 pathways in the brain of *S*. *pneumoniae*-infected mice.

### Blockage of NF-κB p65 and MAPK p38 attenuates B7-H3-amplified inflammatory response and ameliorates B7-H3-exacerbated BBB disruption in S. pneumoniae-infected mice

**Fig 5:**
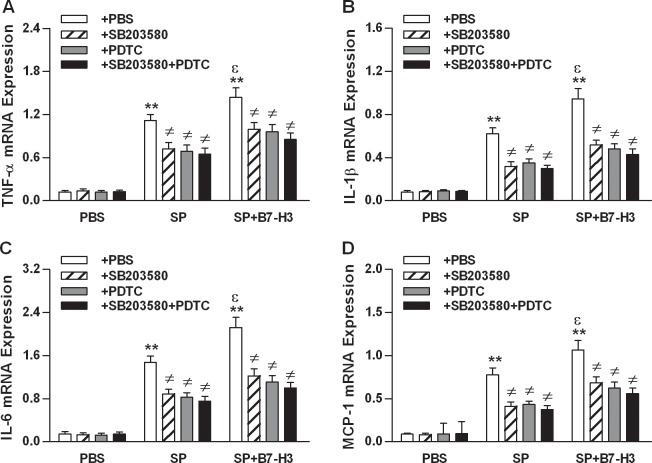
Blockage of NF-κB p65 and/or MAPK p38 attenuates B7-H3-amplified proinflammatory cytokine and chemokine mRNA expression in brain tissues of *S*. *pneumoniae*-infected mice. Mice were challenged with PBS as the control, live *S*. *pneumoniae* (SP), or live *S*. *pneumoniae* plus B7-H3 (SP+B7-H3) 1 hr after mice pretreated with the MAPK p38 inhibitor SB203580, the NF-κB p65 inhibitor PDTC, or their combination (SB203580+PDTC) as described in the Methods. Brain samples were collected at 12 hrs after challenges for detecting mRNA expression of TNF-α (**A**), IL-1β (**B**), IL-6 (**C**), and MCP-1 (**D**) by quantitative real-time PCR. Data are expressed as mean ± SD of five to six mice per time point and represent two separate experiments. **p<0.01 compared with mice treated with PBS, ^≠^*p*<0.05 compared with mice treated with SP alone or SP+B7-H3, ^ε^*p*<0.05 compared with SP alone.

**Fig 6:**
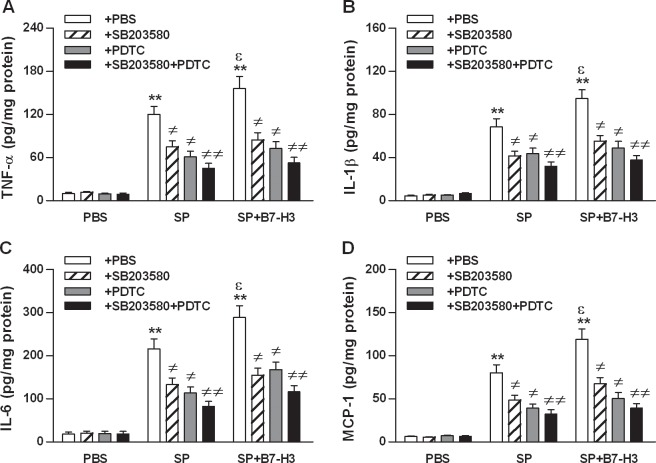
Blockage of NF-κB p65 and/or MAPK p38 attenuates B7-H3-amplified proinflammatory cytokine and chemokine production in brain tissues of *S*. *pneumoniae*-infected mice. Mice were challenged with PBS as the control, live *S*. *pneumoniae* (SP), or live *S*. *pneumoniae* plus B7-H3 (SP+B7-H3) 1 hr after mice pretreated with the MAPK p38 inhibitor SB203580, the NF-κB p65 inhibitor PDTC, or their combination (SB203580+PDTC) as described in the Methods. Brain samples were collected at 18 hrs after challenges for detecting protein levels (**B**) of TNF-α (**A**), IL-1β (**B**), IL-6 (**C**), and MCP-1 (**D**) by ELISA. Data are expressed as mean ± SD of five to six mice per time point and represent two separate experiments. **p<0.01 compared with mice treated with PBS, ^≠^*p*<0.05, ^≠≠^*p*<0.01 compared with mice treated with SP alone or SP+B7-H3, ^ε^*p*<0.05 compared with SP alone.

Having demonstrated that B7-H3 administration strongly augments the activation of TLR2 downstream signaling pathways, NF-κB p65 and MAPK p38, in the brain of *S*. *pneumoniae*-infected mice, we first attempted to determine whether the augmented activation of NF-κB p65 and/or MAPK p38 is responsible for B7-H3-amplified inflammatory response. Significantly increased proinflammatory cytokine and chemokine TNF-α, IL-1β, IL-6, and MCP-1 mRNA expression (Fig 5) and protein levels (Fig 6) in brain homogenates were observed in mice infected with *S*. *pneumoniae* (*p*<0.01 versus mice treated with PBS), which was further augmented in mice challenged with *S*. *pneumoniae* and B7-H3 in combination (*p*<0.05 versus mice challenged with *S*. *pneumoniae* alone) (Fig 5 and Fig 6). Blockage of MAPK p38 *in vivo* with its specific inhibitor SB203580 substantially attenuated B7-H3-augmented TNF-α, IL-1β, IL-6, and MCP-1 expression at both the mRNA level (Fig 5) and the protein level (Fig 6) in the brain of *S*. *pneumoniae*-infected mice (*p*<0.05 versus mice challenged with *S*. *pneumoniae* and B7-H3 in combination). Blockage of NF-κB p65 with its specific inhibitor PDTC caused similar attenuation in B7-H3-augmented TNF-α, IL-1β, IL-6, and MCP-1 production in *S*. *pneumoniae*-infected mice (Fig 5 and Fig 6). Of note, blocking both MAPK p38 and NF-κB p65 with SB203580 and PDTC led to further attenuation at the protein level of B7-H3-augmented proinflammatory cytokines and chemokine in *S*. *pneumoniae*-infected mice (*p*<0.01 versus mice challenged with *S*. *pneumoniae* and B7-H3 in combination) (Fig 6).

**Fig 7:**
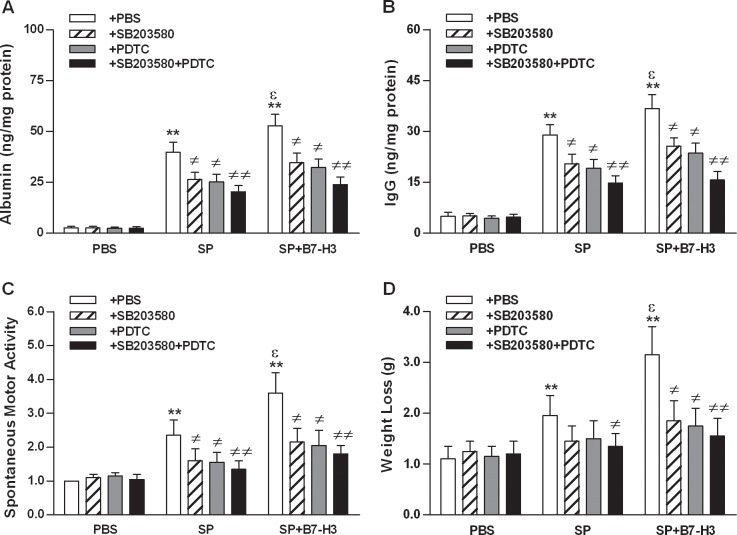
Blockage of NF-κB p65 and/or MAPK p38 ameliorates B7-H3-exacerbated disruption of BBB integrity and severity of disease status in *S*. *pneumoniae*-infected mice. Mice were challenged with PBS as the control, live *S*. *pneumoniae* (SP), or live *S*. *pneumoniae* plus B7-H3 (SP+B7-H3) 1 hr after mice pretreated with the MAPK p38 inhibitor SB203580, the NF-κB p65 inhibitor PDTC, or their combination (SB203580+PDTC) as described in the Methods. Brain samples were collected at 30 hrs after challenges for detecting albumin (**A**) and IgG (**B**) levels using ELISA. The severity of disease status as represented by spontaneous motor activity (**C**) and body weight loss (**D**) was examined 30 hrs after challenges. Data are expressed as mean ± SD of five to six mice per time point and represent two separate experiments. **p<0.01 compared with mice treated with PBS, ^≠^*p*<0.05, ^≠≠^*p*<0.01 compared with mice treated with SP alone or SP+B7-H3, ^ε^*p*<0.05 compared with SP alone.

We next attempted to determine whether the augmented activation of NF-κB p65 and/or MAPK p38 is involved in B7-H3-exacerbated BBB disruption and clinical disease status. Mice challenged with *S*. *pneumoniae* displayed disruption of BBB integrity with significantly increased albumin (Fig 7A) and IgG (Fig 7B) in brain homogenates and showed moderate severity of disease with substantially decreased spontaneous motor activity (Fig 7C) and increased body weight loss (Fig 7D) at 30 hrs after infection (*p*<0.01 versus mice treated with PBS), whereas administration of B7-H3 further aggravated BBB disruption (Fig 7A and 7B) and disease severity (Fig 7C and 7D) in *S*. *pneumoniae*-infected mice (*p*<0.05 versus mice challenged with *S*. *pneumoniae* alone). However, blockage of NF-κB p65 and/or MAPK 38 with their specific inhibitors significantly attenuated B7-H3-exaggerated extravasation of serum albumin and IgG into the brain (Fig 7A and 7B) and substantially ameliorated B7-H3-exacerbated disease severity (Fig 7C and 7D) in *S*. *pneumoniae*-infected mice (*p*<0.05, *p*<0.01 versus mice challenged with *S*. *pneumoniae* and B7-H3 in combination). Collectively, these results strongly indicate that activation of both NF-κB p65 and MAPK p38 pathways is predominantly responsible for B7-H3-amplified inflammatory response and is involved in B7-H3-exacerbated disruption of BBB integrity and severity of disease status during pneumococcal meningitis.

## Discussion

In addition to a well documented role of B7-H3 in regulating Ag-specific T cell-mediated adaptive immune responses [16–19], B7-H3 has been shown to be involved in the innate immune response by acting as a costimulator of innate immunity to amplify inflammatory responses [13,21]. B7-H3 markedly enhanced both the TLR4 agonist LPS- and the TLR2 agonist BLP-stimulated proinflammatory cytokine production in monocytes/macrophages, whereas neutralization of B7-H3 with its blocking mAb MIH 35 significantly attenuated proinflammatory cytokine release and protected mice against LPS-associated lethality [21]. By using a murine model of pneumococcal meningitis, we previously demonstrated that B7-H3 strongly augmented *S*. *pneumoniae*-induced inflammatory response with exacerbated BBB disruption and aggravated brain injury, which occurs in a TLR2-dependent manner as B7-H3 almost completely lost its amplifying action on *S*. *pneumoniae*-induced inflammatory response in TLR2-deficinet mice [13]. In the present study, we further examined the impact of B7-H3 on activation of TLR2 signaling and aimed to clarify the component(s) of TLR2-mediated signal transduction pathways responsible for B7-H3-amplified inflammatory response and subsequent brain damage during experimental pneumococcal meningitis. Administration of B7-H3 resulted in an enhanced MyD88-IRAK immunocomplex formation in *S*. *pneumoniae*-infected mice, but had no augmenting effects on expression of TLR2 and other upstream components including MyD88, IRAK1, TIRAP, and TRAF6. Furthermore, B7-H3 substantially augmented *S*. *pneumoniae*-induced activation of the TLR2 downstream NF-κB p65 and MAPK p38 pathways in the brain of *S*. *pneumoniae*-infected mice. Remarkably, blockage of NF-κB p65 and/or MAPK p38 attenuated B7-H3-amplified inflammatory response with significantly reduced proinflammatory cytokine and chemokine production, and ameliorated B7-H3-exacerbated disruption of BBB integrity and severity of disease status in *S*. *pneumoniae*-infected mice.

TLR2 acts primarily as an innate sensor of gram-positive bacteria such as *S*. *pneumoniae* by detecting their cell wall products including bacterial lipoprotein, peptidoglycan, and lipoteichoic acid [28–32]. Upon engagement with these PAMPs of gram-positive bacteria, TLR2 recruits the adaptor proteins MyD88, IRAK1, and TRAF6 [26–30]. In turn, these adaptor molecules activate the TLR2-mediated downstream signal transduction pathways NF-κB and MAPKs including p38, extracellular signal-regulated kinase 1/2 (ERK1/2), and c-Jun NH2-terminal kinase (c-Jun), which ultimately leads to transcription of the targeted inflammatory genes and production of proinflammatory cytokines and chemokines [26,27,30,31]. Activation of TLR2 is the first step to initiate a cascade of the above mentioned intracellular signal events that eventually trigger the inflammatory response. Previous work has shown that stimulation with bacterial lipoprotein substantially enhances TLR2 expression and blockage of TLR2 strongly attenuates bacterial lipoprotein-stimulated TNF-α and IL-6 production [33,34]. Significantly upregulated TLR2 mRNA expression was also detected in the brain of *S*. *pneumoniae* infection-induced experimental pneumococcal meningitis [11]. In the present study, we observed substantially enhanced TLR2 mRNA expression, but not TLR2 protein expression, in the brain of *S*. *pneumoniae*-infected mice, whereas additional B7-H3 challenge failed to further augment *S*. *pneumoniae*-stimulated TLR2 expression. Surprisingly, neither *S*. *pneumoniae* nor *S*. *pneumoniae* in combination with B7-H3 affected MyD88, IRAK1, TIRAP, and TRAF6 mRNA expression. Of note, infection with *S*. *pneumoniae* or *S*. *pneumoniae* in combination with B7-H3 led to reduced protein expression of IRAK1. Crucially, administration of B7-H3 further enhanced MyD88-IRAK immunocomplex formation in the brain of *S*. *pneumoniae*-infected mice, indicating that B7-H3 amplifies *S*. *pneumoniae*-stimulated activation of TLR2 upstream signaling pathways by augmentation of MyD88-IRAK immunocomplex formation.

NF-κB p65 and MAPK p38 are two common downstream pathways for TLR2 signaling, and activation of NF-κB p65 and/or MAPK p38 is a critical event to trigger transcription of the targeted inflammatory genes and production of proinflammatory cytokines and chemokines [26–31]. In the present study, we tested whether B7-H3 is capable of augmenting *S*. *pneumoniae*-induced activation of NF-κB p65 and MAPK 38, thus amplifying inflammatory responses in *S*. *pneumoniae*-infected mice. We demonstrated that infection of mice with *S*. *pneumoniae* resulted in activation of both NF-κB p65 and MAPK 38 pathways. Of note, the additional challenge with B7-H3 led to further elevations in phosphorylated NF-κB p65 and MAPK p38 in the brain of *S*. *pneumoniae*-infected mice. Having demonstrated the augmenting effect of B7-H3 on *S*. *pneumoniae*-induced activation of NF-κB p65 and MAPK p38, we further addressed whether these two key downstream components of the TLR2-mediated signal transduction pathway is indeed responsible for B7-H3-amplified inflammatory response *S*. *pneumoniae*-infected mice. Remarkably, blockage of NF-κB p65 and/or MAPK p38 with their specific inhibitors strongly attenuated B7-H3-amplified TNF-α, IL-1β, IL-6, and MCP-1 production in the brain of *S*. *pneumoniae*-infected mice, suggesting that B7-H3-induced additional activation of both NF-κB p65 and MAPK p38 is predominantly responsible for B7-H3-amplified inflammatory response during experimental pneumococcal meningitis.

It has been well documented that a persistent and/or amplified activation of inflammatory response with the excessive production of proinflammatory cytokines in the CNS can cause severe damage to the brain and predominantly contributes to a frequently unfavorable outcome during the development of pneumococcal meningitis [8,10,14]. We therefore asked whether B7-H3 augmented NF-κB p65 and/or MAPK p38 activation and subsequently amplified proinflammatory cytokine and chemokine production are responsible for the observed exacerbation of BBB disruption and severity of disease status in mice challenged with *S*. *pneumoniae* in combination with B7-H3. We demonstrated that administration of B7-H3 further aggravated BBB disruption with significantly increased albumin and IgG in the brain, and intensified disease severity with substantially decreased spontaneous motor activity and increased body weight loss in *S*. *pneumoniae*-infected mice. Remarkably, blockage of either NF-κB p65, MAPK 38 or both with their specific inhibitors significantly attenuated B7-H3-exaggerated extravasation of serum albumin and IgG into the brain and substantially ameliorated B7-H3-exacerbated disease severity in *S*. *pneumoniae*-infected mice, indicating that B7-H3 augmented activation of both NF-κB p65 and MAPK p38 pathways and subsequently amplified inflammatory response is predominantly responsible for B7-H3 exacerbated disruption of BBB integrity and severity of disease status during pneumococcal meningitis.

Taken together, in this study we demonstrate that administration of B7-H3 results in enhanced formation of MyD88-IRAK immunocomplex and augmented activation of TLR2 downstream NF-κB p65 and MAPK p38 pathways in the brain of *S*. *pneumoniae*-infected mice. We have further shown that blockage of NF-κB p65 and/or MAPK p38 attenuates B7-H3-amplified inflammatory response and ameliorates B7-H3-exacerbated BBB disruption and brain damage in *S*. *pneumoniae*-infected mice, suggesting that targeting NF-κB p65 and/or MAPK p38 may represent a potential therapeutic option for amelioration of overwhelming inflammatory response-associated brain injury during pneumococcal meningitis.

## Abbreviations

BBB: blood-brain barrier
BLP: bacterial lipoprotein
c-Jun: c-Jun NH2-terminal kinase
CNS: central nervous system
CSF: cerebrospinal fluid
ERK1/2: extracellular signal-regulated kinase 1/2
IRAK: IL-1 receptor-associated kinase
LPS: lipopolysaccharide
MAPK: mitogen-activated protein kinase
MyD88: myeloid differentiation factor 88
NF-κB: nuclear factor-kappaB
PAMP: pathogen-associated molecular pattern
PRR: pattern recognition receptors
sB7-H3: soluble B7-H3 (sB7-H3)
TIRAP: toll-interleukin 1 receptor domain containing adaptor protein
TLR: toll-like receptor
TRAF6: TNF receptor-associated factor
